# Association of physical activity and rest-activity rhythm with sustained attention and melatonin among community-dwelling older adults

**DOI:** 10.1186/s11556-026-00413-1

**Published:** 2026-04-18

**Authors:** PinShiuan Lee, Yi-Ling Chen, Yun-Chieh Yang, Tomohide Kubo, Wan-Ju Cheng

**Affiliations:** 1 National Health Research Institutes, Miaoli, Taiwan; 2 National Institute of Occupational Safety and Health, Tokyo, Japan; 3 China Medical University, Taichung, Taiwan

**Keywords:** Sustained attention, Rest-activity rhythm, Actigraphy, Melatonin, Sleep

## Abstract

**Background:**

Physical activity is a critical determinant of health in aging populations. Although the health benefits of exercise are well established, limited research has examined how physical activity patterns and rest-activity rhythms influence biological rhythms and cognitive performance. This study aimed to investigate the relationships between physical activity, rest-activity rhythm, melatonin, and sustained attention in older adults.

**Methods:**

We recruited 147 community-dwelling adults aged ≥ 60 years, who were instructed to wear wrist accelerometers for 14 consecutive days. From the accelerometry data, we derived measures of daytime physical activity (most active 10 h), the distribution of moderate-to-vigorous activity, and 24 h rest-activity rhythm parameters, including interdaily stability, amplitude, autocorrelation coefficient, and relative amplitude. Salivary melatonin was collected at home, and dim-light melatonin onset was calculated. Sustained attention was assessed using a 3-minute smartphone-based psychomotor vigilance task.

**Results:**

Greater amounts of daytime physical activity and a higher proportion of high-intensity activity were associated with more robust rest-activity rhythms, as evidenced by higher interdaily stability, relative amplitude, 24 h autocorrelation, and activity amplitude. In turn, higher interdaily stability was associated with greater peak melatonin levels (β = 39.38; 95% CI = 6.02 to 72.73). In the psychomotor vigilance task, higher relative amplitude was associated with fewer lapses (β = -32.61; 95% CI = -55.60 to -9.62).

**Conclusions:**

Physical activity is associated with a stronger rest-activity rhythm, which further associated with and better health outcomes in older adults. Future studies are warranted to clarify the interactions between physical activity, rest-activity patterns, and biological rhythms.

## Introduction

Physical activity helps preserve quality of life and prevent the development of chronic diseases in later life [1]. While its health benefits are widely acknowledged, physical activity varies considerably in terms of type, intensity, and accessibility. To systematically evaluate the health effects of physical activities and provide practical recommendations for older adults [2], researchers have employed quantitative and qualitative assessments for it (e.g., metabolic equivalents and oxygen consumption). Additionally, objective tools—such as accelerometers—have been developed to track physical activity more accurately. Accelerometer-derived activity indicators include the amount and distribution of activities, the latter indicating how different intensities contribute to total daytime activity [3]. For example, a combination of a low level of moderate-to-vigorous physical activity (MVPA) and a high level of sedentary time is associated with a high risk of mortality in older adults [4]. The evenness of activity distribution can be evaluated using the Gini index and the intensity gradient [3, 5], which reflect the time accumulated at different activity intensities and provide delicate information beyond total physical activity volume. Few studies have used these indicators to evaluate activity distribution, which has been found to be associated with physical function [6, 7].

In addition to the amount and distribution of physical activity, a growing body of research has explored the relationship between physical activity and circadian rhythm. Notably, rest-activity rhythms and biological circadian rhythms are bidirectional; the underlying biological clock dictates rest-activity patterns, while the timing of physical activity acts as a critical zeitgeber, providing the necessary cues to entrain the endogenous biological clock [8]. Physical activity has been associated with more robust rest-activity rhythm in older adults [9]. This relationship may be explained by biological mechanisms such as skeletal muscle clock modulation [10]; additionally, a routine of regular exercise is often associated with a more robust rest-activity rhythm. Rest-activity rhythm robustness can be evaluated using regularity indicators such as interdaily stability (IS) [11] and 24 h autocorrelation coefficients [12], as well as measures of activity amplitude, including relative amplitude (RA) [11] and amplitude derived from cosinor analysis [13]. A more robust rest-activity rhythm is associated with better health. Cohort studies have revealed that decreased RA is associated with all-cause mortality and multimorbidity [14–16]. Lower IS and reduced 24 h autocorrelation have been associated with an unfavorable cardiometabolic biomarker profile in men [17].

Among the various biological markers of the human circadian system, melatonin secretion is a principal marker due to its entrainment by the light-dark cycle. Dim light melatonin onset (DLMO) is considered the gold-standard measurement for internal circadian phase. Generally, older adults exhibit a phase-advanced circadian rhythm and a reduced melatonin amplitude compared to younger individuals [18, 19]. While physical exercise may induce a short-term rise in plasma melatonin [20], nocturnal exercise has been shown to induce a phase-delay shift in the circadian rhythm—as measured by DLMO—in older adults [8]. Understanding how the volume and rhythmic patterns of activity influence biological rhythms in this population may offer insights for developing tailored physical activity recommendations; however, this area remains significantly underexplored.

Cognitive impairment and dementia represent major threats to the functional independence of older adults. Systemic-analysis studies concluded that leisure time MVPA, mostly collected by questionnaires, was associated with executive function and memory; but evidence for attention function was inconclusive [21, 22]. Meta-analysis studies for intervention trials observed an association between physical activity and executive function [23], which further facilitates attention performance in older adults [24, 25]. Additionally, disrupted rest-activity rhythm has been observed to be associated with cognitive impairment and dementia [26, 27]. Given the importance of sustained attention to the daily safety and learning of older adults, identifying the specific characteristics of physical activity and rest-activity rhythms that associate with sustained attention would facilitate the design of more targeted interventions.

**Fig. 1:**
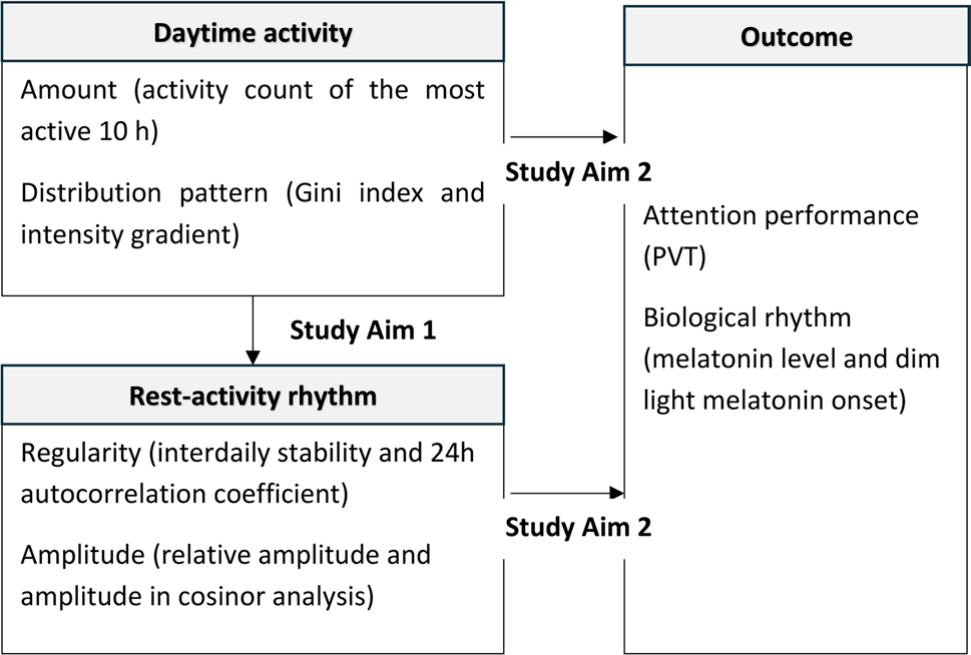
Research framework. Daytime physical activity was characterized by amount, and the Gini index and the intensity gradient as distribution metrics. Rest-activity rhythms were assessed through regularity (interdaily stability and 24 h autocorrelation coefficient) and amplitude (relative amplitude and cosinor-derived amplitude). These daytime activity and rest-activity rhythm parameters enabled examination of the relationships among physical activity, attention performance, and circadian biological rhythms.

Despite the well-documented benefits of physical activity for cognitive health in older adults, empirical evidence regarding the relationship between physical activity and rest-activity rhythm, as well as their links with melatonin secretion and cognitive function, remains limited. As shown in Fig. 1, this study aims to examine: (1) the associations between objectively measured physical activity levels and rest-activity rhythms, and (2) the relationship of physical activity levels and rest-activity rhythms with melatonin secretion and cognitive function, specifically sustained attention, in community-dwelling older adults. We hypothesize that increased physical activity is linked to more robust rest-activity rhythms, and that both associate to higher melatonin secretion and enhanced sustained attention in older adults.

## Methods

### Participants

The study recruited 147 community-dwelling adults aged 60 years and older through advertisements on community billboards. Exclusion criteria encompassed (1) serious neurological or psychiatric diagnoses, (2) physical limitations impairing independent ambulation, and (3) diagnoses of sleep disorders or current use of hypnotics. Participants were instructed to wear a wrist-worn accelerometer for 14 consecutive days to record physical activity. Attention performance was evaluated on the first day of study using the Psychomotor Vigilance Task (PVT), a test of sustained attention that measures the speed and consistency of responses to visual stimuli. Melatonin levels were evaluated on the 14th day through an at-home saliva-sampling protocol. All participants provided written informed consent, and the study was approved by the Research Ethics Committee of the [concealed for review].

### Accelerometry data collection

Participants were instructed to wear a wrist actigraphy device (MotionWatch8, CamNtech Inc, England, UK) on their non-dominant wrist and maintain a sleep diary for 14 consecutive days. Sleep periods and non-wear intervals were manually marked using MotionWatch 8 software (MotionWare v1.2.28) according to the sleep diaries. Non-wear periods were identified by the longest continuous period of zero activity between 07:00 and noon, and 13:00 and 22:00 [28]. On average, non-wear periods accounted for 2.19% of the total recording period. Because sleep is closely related to physical activity and rest-activity rhythms, we extracted sleep indicators—including sleep onset latency, total sleep time, wake after sleep onset, time in bed, and sleep efficiency—using MotionWare software.

### Accelerometry variables: physical activity

Activity counts were recorded in 15-second epochs. To quantify physical activity levels, non-parametric methods were applied to derive activity counts of the most active 10 h (M10) in 24 h and activity counts of the least active 5 h (L5) in 24 h. Activity counts of M10 and L5 were averaged over 14 days. Additionally, weekly hours spent in vigorous and moderate activity were derived from the MotionWare [29, 30].

Regarding the activity distribution of activity within MVPA, we estimated the Gini index and intensity gradient. Following Landry et al., MVPA was identified as ≥ 562.5 activity counts per minute in this study [29]. The Gini index is derived from the Lorenz curve, which represents a theoretical line of equality where activity is distributed uniformly across bouts [3]. In this study, the Gini index within MVPA quantifies how the distribution of activity deviates from perfect equality. Values range from 0 to 1, reflecting the degree of variability in activity bout lengths [31]. Values close to zero indicate that activity is accumulated in bouts of similar duration, in other words, an equal contribution of all bout lengths to total activity time. Conversely, higher values indicate greater inequality in bout length, typically suggesting that total activity time is dominated by a few long bouts. The intensity gradient describes the distribution of activity intensity across all movement intensities by modeling the negative curvilinear relationship between intensity and the time accumulated. A lower (more negative) value indicates a steeper decline in accumulated time as intensity increases, reflecting relatively little time spent at higher intensity levels. Conversely, a less negative gradient indicates a flatter distribution, signifying a more accumulation of time at higher intensities. The intensity gradient was calculated using R-package GGIR (version 2.10-1) [6].

### Accelerometry variables: rest-activity rhythm

Rhythm regularity was assessed using IS and 24 h autocorrelation, while rhythm magnitude was captured via RA and cosinor-derived amplitude. IS reflects how consistent the circadian rest-activity rhythm is across multiple days. It is calculated by dividing the variance of the mean 24-h activity pattern by the total variance of activity across the recording period [11], with a range of 0 to 1 where a value of 0 indicates a total lack of rhythm and a value of 1 indicates a perfectly stable rhythm. The 24 h autocorrelation quantifies the average correlation between activity counts in 1-minute epochs separated by a lag of 24 h (1440 min) [3]. The 24 h autocorrelation coefficient ranges from 0 to 1, with values closer to 1 indicating a strong correlation of activity at the same hour across days.

For rhythm amplitude assessment, RA was calculated as the difference between the average activity level in the M10 and L5 periods. To remove sensitivity to overall activity level, the difference was divided by the sum of the L5 and M10 [32]. A higher RA indicates a more robust (i.e., favorable) rest-activity rhythm. Cosinor analysis was conducted with the cosinor and cosinor2 packages in R 4.3.0, assuming a known period synchronized to a 24-h cycle, when mesor, amplitude, and acrophase components were computed [33].

### Melatonin level and DLMO

The salivary melatonin test for DLMO in older adults has been validated against serum melatonin DLMO measurements [34]. Participants were instructed to provide saliva samples for melatonin in their home environment under dim light conditions (< 50 lx). They were required to maintain clean lips and abstain from caffeine, bananas, alcoholic beverages, and cigarettes during the collection period [35, 36]. Saliva samples were collected every 30 min from 18:00 until 1 h after habitual sleep time on the final study date, with participants refraining from food and drink for 10 min before collection. DLMO was determined based on the two standard deviation threshold method, which uses the average of the first three melatonin data points plus two standard deviations of the same three points [37]. The peak melatonin level was identified among all collected saliva samples.

### The Psychomotor Vigilance Task (PVT)

Attention was evaluated with a smartphone-based 5-minute PVT [38]. The PVT has been widely used to assess sustained attention through reaction time, lapses, and errors [39], and has been adapted into a smartphone-based application for easy use in community settings [38]. A random inter-stimulus interval of 2 to 10 s was used. The number of lapses (a response of longer than 0.5 s) and errors were counted, along with the reaction time (sec), for each 5-min test.

### Self-administered questionnaires

Age and gender information were self-reported by participants. Additional data collected included body height, body weight, any diagnosed chronic diseases, smoking, alcohol drinking, sleep problems, and marital status. Mood symptoms were assessed using the 5-item Brief Symptom Rating Scale (BSRS-5) [40].

### Statistical analyses

Study participants were classified as having a high-amount of activity or a low-amount, based on the median of M10 physical activity counts (16,280 activity counts). Descriptive statistics were conducted using Chi-square and Mann-Whitney tests to compare demographic characteristics, rest-activity rhythm indicators, physical activity indicators, sleep parameters, and PVT performance between the two groups. The association between physical activity and rest-activity rhythm indicators was examined using multiple linear regression analysis. Separate independent models were constructed for each physical activity indicator, adjusted for age, gender, body mass index (BMI), smoking, drinking behaviors, chronic illness, and mood symptoms. M10 activity count was log-transformed and included as a control variable for models estimating the effect of the Gini index and intensity gradient on rhythm indicators. We further examined the association of physical activity indicators and rhythm indicators with biological rhythm variables (DLMO and melatonin) and PVT performance using linear regression analysis. The models were adjusted for age, gender, BMI, smoking, drinking behaviors, chronic illness, mood symptoms, and total sleep time. To account for multiple testing across the six physical activity and rhythm indicators, a Bonferroni-corrected p-value of 0.008 was used as the threshold for statistical significance. The statistical analyses were performed using SAS 9.4 (SAS Institute, Cary, NC, USA).

## Results

### Participant description

Table 1 presents demographic characteristics by groups dichotomized by daytime physical activity level (M10) in old adults. The two groups were similar in age, gender, and health behaviors; however, participants in the high-activity group were less likely to be married compared to those in the low-activity group (62.2% vs. 79.5%). The high-activity group displayed higher IS (0.62 vs. 0.44, *p* < 0.001) and RA (0.88 vs. 0.83, *p* = 0.001) compared to the low-activity group. Sleep parameters, DLMO, and attention test performances were similar between the two groups.

**Table 1:** Group descriptive statistics by physical activity amount in community-dwelling older adults

	High activity (*N* = 74)	Low activity (*N* = 73)	*p* ^a^
	Mean (SD)	Mean (SD)	
Demographics			
Age (years)	68.70 (5.24)	70.42 (6.43)	0.135
Gender, male (N, %)	25 (33.78)	37 (50.68)	0.056
Marital status, married (N, %)	46 (62.16)	58 (79.45)	0.034
Body mass index (kg/m^2^)	23.57 (3.36)	24.37 (3.04)	0.151
Smoke, yes (N, %)	8 (10.81)	7 (9.59)	1.000
Drink, yes (N, %)	8 (10.81)	9 (12.33)	0.976
Chronic Illness, yes (N, %)	52 (70.27)	55 (75.34)	0.613
BSRS-5 score	1.89 (2.22)	2.30 (3.12)	0.449
Rest-activity rhythm indicators			
Interdaily stability	0.62 (0.15)	0.44 (0.10)	**<** ** 0.001**
Relative amplitude	0.88 (0.07)	0.83 (0.11)	0.001
24 h autocorrelation coefficient	0.40 (0.12)	0.34 (0.14)	0.002
Amplitude in cosinor analysis	37.87(13.41)	21.91 (8.73)	**< **0.001
Acrophase from cosinor analysis (hh: mm)	13:15 (01:49)	12:58 (01:52)	0.625
Physical activity indicators			
Vigorous activity (total hours/weekly)	3.42 (2.31)	1.64 (1.24)	**< **0.001
Moderate activity (total hours/weekly)	12.22 (5.62)	6.62 (3.03)	**<** ** 0.001**
Gini Index	0.15 (0.04)	0.16 (0.07)	0.402
Intensity gradient	-1.17 (0.31)	-1.18 (0.40)	0.971
Sleep indicators			
Total sleep time (h)	5.92 (1.10)	6.32 (1.15)	0.053
Sleep efficiency (%)	78.19 (10.63)	80.45 (10.52)	0.185
Sleep onset latency (min)	25.14 (33.44)	22.78 (26.03)	0.538
Wake after sleep onset (min)	67.46 (31.28)	64.66 (37.46)	0.298
Bedtime (hh: mm)	22:32 (01:22)	22:17 (01:13)	0.140
Get-up time (hh: mm)	06:08 (01:07)	06:12 (01:03)	0.455
Melatonin			
Peak melatonin level (pg/ml)	20.51 (35.90)	18.28 (23.75)	0.815
Dim light melatonin onset (hh: mm)	20:32 (01:16)	20:13 (01:11)	0.168
Psychomotor Vigilance Task			
Mean reaction time (ms)	668.41 (1018.07)	508.14 (192.19)	0.547
Number of lapses	10.66 (13.77)	11.22 (11.64)	0.291
Number of errors	8.62 (28.53)	10.62 (32.95)	0.141

*Abbreviations*: *BSRS-5* 5-item Brief Symptom Rating Scale

^a^*P*-values comparing the two groups were calculated using the Mann-Whitney test and chi-square tests

### Associations between physical activity and rest-activity rhythm indicators

The association between physical activity and rest-activity rhythm indicators, that is IS, RA, 24 h autocorrelation coefficient, and activity amplitude from cosinor analysis were examined using multiple regression analysis adjusted for age, gender, BMI, smoking, drinking behaviors, chronic illness, and mood symptoms (Table 2). Log-transformed M10 was associated with a more robust rest-activity rhythm, as indicated by its positive correlation with higher IS (β = 0.20; 95% CI = 0.16 to 0.24), RA (β = 0.08; 95% CI = 0.05 to 0.11), 24 h autocorrelation coefficient (β = 0.07; 95% CI = 0.03 to 0.12), and amplitude from cosinor analysis (β = 19.4; 95% CI = 16.05 to 22.77). Hours spent in moderate and vigorous activities were also positively associated with rest-activity rhythm indicators. In addition to activity amount, we further examined the association between MVPA distribution and rest-activity rhythm indicators. In these models, logM10 was included as a covariate to adjust for its potential confounding effect on the association between activity distribution and rest-activity rhythms. A higher Gini index (uneven activity distribution) was associated with lower IS, higher 24 h autocorrelation coefficient, and higher amplitude from cosinor analysis. The intensity gradient was positively associated with RA and negatively associated with IS, suggesting that more high-intensity activities are associated with a clearer distinction between active and inactive periods, but with a lower rhythm regularity.

**Table 2:** Association between physical activity and rest-activity rhythm indicators. All physical activity indicators were included in independent models, and were adjusted for age, gender, body mass index, smoking, drinking behaviors, chronic illness, and mood symptoms. M10 activity count was log-transformed and included as a control variable for models estimating the effect of the Gini index and intensity gradient.

	Outcome: interdaily stability			Outcome: relative amplitude		
Physical activity indicators	β	(95% CI)	*P*	β	(95% CI)	*P*
M10 activity count	0.198	(0.158, 0.238)	< 0.001	0.075	(0.045, 0.105)	< 0.001
Vigorous activity	0.028	(0.016, 0.040)	< 0.001	0.009	(0.001, 0.017)	0.020
Moderate activity	0.016	(0.012, 0.020)	< 0.001	0.005	(0.002, 0.008)	0.003
Gini index	-0.533	(-0.865, -0.202)	0.002	0.111	(-0.143, 0.364)	0.390
Intensity gradient	-0.086	(-0.139, -0.033)	0.002	0.045	(0.005, 0.085)	0.029

	Outcome: 24 h autocorrelation coefficient			Outcome: amplitude		
Physical activity indicators	β	(95% CI)	*P*	β	(95% CI)	*P*
M10 activity count	0.072	(0.027, 0.116)	0.002	19.412	(16.049, 22.774)	< 0.001
Vigorous activity	0.012	(0.001, 0.023)	0.041	4.115	(3.202, 5.028)	< 0.001
Moderate activity	0.005	(0.001, 0.009)	0.023	1.474	(1.104, 1.843)	< 0.001
Gini index	0.520	(0.156, 0.885)	0.006	48.537	(21.162, 75.912)	0.001
Intensity gradient	0.030	(-0.030, 0.090)	0.319	2.182	(-2.383, 6.746)	0.346

*Abbreviations*: *M10* Average activity during the most active 10-h period

### Associations of physical activity and rest-activity rhythms with melatonin and attention

We further examined the relationship of physical activity and rest-acivity rhythm indicators with DLMO and peak melatonin level (Table 3). IS was positively associated with peak melatonin level (β = 39.38; 95% CI = 6.02 to 72.73) but not with DLMO. No significant associations were observed between physical activity or rhythm indicators and melatonin levels after Bonferroni adjustment for multiple comparisons. Regarding PVT performance (Table 4), RA was negatively associated with the number of lapses (β = -32.61; 95% CI = -55.60 to -9.62), while IS was positively associated with mean reaction time (β = 956.07; 95% CI = 160.45 to 1751.70). The association between RA and lapses remained significant after Bonferroni adjustment.

**Table 3:** Association of physical activity with DLMO and melatonin. The models were adjusted for age, gender, body mass index, smoking, drinking, total sleep time, chronic illness, mood symptoms, and total sleep time. The physical activity indicators were examined in the regression models one at a time

Physical activity and rest-activity rhythm	Outcome: DLMO (hh: mm)			Outcome: peak melatonin level (pg/ml)		
	β	(95% CI)	*P*	β	(95% CI)	*P*
M10 (high vs. low)	0.340	(-0.072, 0.752)	0.105	-0.466	(-10.873, 9.942)	0.930
M10 (continuous)	0.272	(-0.161, 0.705)	0.216	-0.749	(-11.423, 9.925)	0.890
Relative amplitude	0.781	(-1.411, 2.972)	0.483	-0.708	(-55.448, 54.032)	0.980
Interdaily stability	1.028	(-0.374, 2.431)	0.149	39.375	(6.024, 72.726)	0.021
24 h autocorrelation coefficient	1.250	(-0.303, 2.803)	0.114	37.210	(-1.024, 75.445)	0.056
Amplitude	0.008	(-0.008, 0.023)	0.328	0.332	(-0.041, 0.705)	0.081

*Abbreviations*: *M10* Average activity during the most active 10-h period, *DLMO* dim light melatonin onset

**Table 4:** Association of physical activity and rest-activity rhythm with psychomotor vigilance task performance. The models were adjusted for age, gender, body mass index, smoking, drinking behavior, total sleep time, chronic illness and mood symptoms, and total sleep time. The variables were examined in the regression models one at a time. M10 activity count was log-transformed for analysis

Physical activity and rest-activity rhythm	Outcome: number of lapses			Outcome: number of errors			Outcome: mean reaction time (ms)		
	Β	(95% CI)	*P*	β	95% CI	*P*	β	95% CI	*P*
M10 (high vs. low)	-0.522	(-5.016, 3.972)	0.819	-0.444	(-11.176, 10.289)	0.935	105.98	(-141.823, 353.784)	0.399
M10 (continuous)	-2.526	(-7.116, 2.065)	0.279	-0.137	(-11.145, 10.872)	0.981	164.98	(-88.326, 418.286)	0.200
Relative amplitude	-32.607	(-55.597, -9.618)	0.006	-3.810	(-60.260, 52.640)	0.894	249.528	(-1056.578, 1555.634)	0.706
Interdaily stability	7.732	(-6.897, 22.361)	0.298	-13.117	(-48.119, 21.885)	0.460	956.071	(160.446, 1751.697)	0.019
24 h autocorrelation coefficient	2.303	(-14.427, 19.033)	0.786	-10.251	(-50.175, 29.672)	0.613	550.130	(-370.206, 1470.466)	0.239
Amplitude	-0.069	(-0.232, 0.093)	0.400	-0.085	(-0.474, 0.303)	0.664	5.950	(-3.000, 14.900)	0.191

*Abbreviations*: *M10* Average activity during the most active 10-h period

## Discussion

In this study, we observed that a higher daytime activity (M10) amount was associated with more robust rest-activity rhythm. Regarding MVPA distribution, more time accumulated at higher intensities (higher intensity gradient) was associated with a higher RA, 24 h autocorrelation coefficient, and rhythm amplitude in cosinor analysis. In turn, a higher RA was associated with fewer lapses in PVT tests.

### Physical activity amount as a predictor of rest-activity rhythm robustness

This positive association between daytime activity (M10) amount and a more robust rest-activity rhythm is consistent with previous studies in older adults [9], individuals with early-onset dementia [41], and cancer patients [42]. Notably, sleep parameters were similar between the high- and low-activity groups, suggesting that the observed rhythm stability was not primarily driven by sleep quality or duration. Nevertheless, due to the cross-sectional design of this study, the causal inference is unclear. Older adults who are more physically active may be healthier than those who are inactive and better able to maintain a regular lifestyle. Vigorous physical activity increases the rest-activity rhythm amplitude and is inherently linked to rhythm stability and robustness [43]. Additionally, physical activity serves as a potent zeitgeber that stabilizes the rest-activity rhythm through the modulation of the skeletal muscle clock, body temperature, and hormonal pathways [10, 44]. Conversely, those who follow a more regular rest-activity schedule may place greater value on their health and therefore engage in more physical activity [9].

For MVPA distribution, we observed that a more uneven distribution (higher Gini index) and more accumulation in high intensity activities (higher intensity gradient) were associated with greater overall activity amplitude and activity distinction between day and night (higher RA). The association persisted after adjustment for total daytime activity (M10) and total sleep time. Given that RA captures the difference between daytime and nighttime activity, this finding implies that high-intensity physical activity may contribute to reduced nocturnal activity, reflecting better sleep quality. Regarding rhythm regularity indicators, namely IS and 24 h autocorrelation coefficients, the findings appear paradoxical. While 24 h autocorrelation reflects the consistency of activity levels at the same time each day, its positive association with high-intensity activity suggests that such exercise is scheduled at consistent daily times. However, if high-intensity activity is not performed daily, the variability across days may reduce IS. This finding suggests that IS should be interpreted with caution, taking into account the intensity and timing of physical activity. The association between IS and cognitive decline, as well as the risk of Alzheimer’s disease, is inconsistent in the literature [45–48]. These contradictory findings suggest that IS may be influenced by factors beyond endogenous circadian biology, including factors such as routine monotony, overall activity volume, and the temporal distribution of activity.

### Interdaily stability as a predictor of higher melatonin levels

In this study, higher IS was associated with increased peak melatonin levels; however, this association did not persist after Bonferroni correction. No significant association was found between IS and DLMO. DLMO occurs earlier in older participants compared to younger individuals, and melatonin levels decline significantly after age 70 [49]. The relatively low level of melatonin in older adults, combined with potentially non-standardized light exposure control in the home setting during saliva collection, may have resulted in the null associations observed in this study. Evidence suggests that physical exercise may induce a short-term, transient rise in plasma melatonin, but the response depends on baseline melatonin levels and the timing of exercise in relation to melatonin secretion, and it appears to be attenuated with regular training [20]. Given that melatonin is a potent antioxidant [50], the potential health benefits of enhancing rhythm regularity via this pathway make this worthy of further investigation. Nevertheless, it is also possible that individuals with higher plasma melatonin may find it easier to maintain a regular rest-activity rhythm. In this study, the total amount of daytime physical activity was not associated with either DLMO or melatonin levels. Notably, data regarding the timing of physical activity, a key zeitgeber influencing biological rhythms [20], was absent in this study. Future research including the timing of activity, rather than focusing solely on total volume and rhythm regularity, may reveal more nuanced associations with biological circadian indicators.

### Associations between rest-activity rhythm characteristics and attentional performance

Our findings revealed an intriguing picture of attention performance. While high RA was associated with fewer lapses, higher IS was associated with longer reaction time (prior to Bonferroni correction). The reaction time in our sample was around 430–445 ms, which was longer than in young adults testing the same protocol [38]. Empirical evidence indicates that compared to younger adults, older adults demonstrated a longer response time accompanied by superior accuracy metrics compared to their younger counterparts [51, 52], reflecting that older adults adopt approaches characterized by increased accuracy, with a trade-off of slower reaction. In this study, older adults with a more robust rest-activity rhythm likely demonstrated a speed-accuracy tradeoff, characterized by slower responses but improved sustained attention. Researchers suggest that age-related changes in cognitive control, while traditionally viewed as examples of decline, may confer adaptive advantages in creative problem-solving and pattern recognition [53, 54]. The longer reaction times observed in individuals with more regular rest-activity rhythm may indicate a strategic cognitive preference for comprehensive information processing rather than rapid response. In this study, activity amplitude estimated via cosinor analysis and 24-hour autocorrelation was not associated with PVT performance. This lack of association may be explained by the temporal misalignment between a longitudinal 14-day actigraphy period and a single-point PVT assessment, which may fail to capture the transient fluctuations in attentional capacity. Nevertheless, our findings suggest that nonparametric measures, such as RA and IS, demonstrated stronger association with PVT performance compared to indicators that rely on model-based assumptions, such as the sinusoidal constraints of cosinor analysis.

### Strengths and limitations

This study employed a community setting and explored older adults with varying physical activity patterns using objective continuous accelerometry measurements for activity. However, there are limitations of this study. First, the study participants were restricted to a comparatively small sample size and comprised relatively healthy older adults, which restricts the generalizability of our findings. Second, cognitive function was measured using a single tool, omitting the impact of physical activity and circadian rhythm on a broader aspect of cognitive functions. Third, the study design was cross-sectional, which prevents causal inference between physical activity, circadian rhythm, and attention performance. There may exist individual (e.g., genetic traits) and environmental factors (e.g., light exposure and social activity patterns) that determined study participants’ rest-activity rhythm, physical activity, and cognitive performance.

## Conclusions

Among older adults, a higher amount of daytime physical activity and engagement in more high-intensity activities were associated with a more robust rest-activity rhythm. In turn, rhythm robustness was linked to higher melatonin levels and better sustained attention performance. These findings suggest a potential pathway, via rest-activity rhythm, linking physical activity to health outcomes in older adults. Future studies are warranted to examine how different types and timing of physical activity influence both rest-activity and biological rhythms.

## Data Availability

The data that support the findings of this study are available from the corresponding author, upon reasonable request.

## Abbreviations

PVT: Psychomotor vigilance task
IS: Interdaily stability
RA: Relative amplitude
MVPA: Moderate-to-vigorous physical activity
DLMO: Dim light melatonin onset
BMI: Body mass index
M10: Most active 10 h
L5: Least active 5 h
